# Food Insecurity Among University Students: Evidence from Three Cross-Sectional Surveys, 2020–2024

**DOI:** 10.3390/nu18193124

**Published:** 2026-09-23

**Authors:** Putu Novi Arfirsta Dharmayani, Seema Mihrshahi

**Affiliations:** 1 School of Health Sciences and Nursing, Faculty of Medicine, Health and Human Sciences, Macquarie University, Macquarie Park, NSW 2109, Australia; 2 Sydney School of Public Health, Faculty of Medicine and Health, The University of Sydney, Camperdown, NSW 2050, Australia

**Keywords:** food insecurity, university students, COVID-19, higher education, Australia

## Abstract

**Background/Objectives:** Food insecurity among university students is a complex yet often overlooked issue. This issue has been exacerbated by inflation, higher living costs, and the COVID-19 pandemic. This study aimed to examine the change in the prevalence of food insecurity among Australian university students during and after the COVID-19 pandemic, and to identify its sociodemographic associations. **Methods:** We conducted three online cross-sectional surveys using a convenience sample of students from an Australian university from 2020 to 2024 (n = 1010). Food insecurity status was assessed using the six-item food security survey module. We analysed the data using bivariate statistics and multivariable logistic regression to assess the associations between food insecurity and sociodemographic variables. **Results:** The prevalence of food insecurity has remained consistently high, at around 40%, since the pandemic, with a rising proportion reporting very low food security from 7.6% in 2020 to 16.5% in 2024. Moreover, international students are disproportionately affected, facing higher levels of food insecurity than domestic students. This study also suggests that household size emerged as a predictor factor of food insecurity, but its association differed by student status. **Conclusions:** Future research should explore social determinants of health, such as housing situation, to better understand their relationship with food insecurity among university students. Our findings highlight the importance of routine and systematic monitoring of food security in higher education settings and proactive efforts should prioritise vulnerable students by providing appropriate resources and advocating for effective food policies to address food insecurity.

## 1. Introduction

Approximately 2.3 billion people experienced moderate or severe food insecurity in 2024, which is around 335 million more than in 2019 [1]. If current trends continue, the United Nations 2030 target “Zero Hunger” will not be met. According to a Delphi consensus study on food security in high-income countries, food insecurity can be defined as the inability to consistently access, obtain, and afford adequate safe, nutritious, and culturally appropriate food that meets dietary needs and preferences in socially acceptable ways [2].

Although food insecurity is frequently overlooked in high-income countries, evidence indicates a rising prevalence of moderate-to-severe food insecurity in these settings. The latest national-level reports indicate that more than 10% of households in the United Kingdom (UK) [3], the United States (US) [4], and Australia [5] experienced food insecurity, with over 5% experiencing moderate-to-severe food insecurity. Thus, data on food insecurity levels is becoming increasingly important for assessing food insecurity across populations due to a worsening of the food and nutrition security crisis, including in high-income countries [6]. After the US Department of Agriculture terminated its Household Food Security Reports in September 2025 [7], Canada is now the only country that conducts regular population-level food security monitoring [6].

In a study of 34 high income countries, young people were shown to have a higher burden of moderate to severe food insecurity, particularly if they live alone [8]. Moreover, evidence from a global study suggests food insecurity increases with age during adolescence but declines across adulthood, and adult immigrants have a higher likelihood of experiencing food insecurity [9]. These age- and migration-related patterns underscore the importance of monitoring food insecurity in settings where a large number of young people and migrants are prevalent, such as universities. The COVID-19 pandemic intensified food insecurity among university students, turning a long-standing issue into a widespread crisis [10,11,12,13]. In Australia, the prevalence of students experiencing food insecurity during the pandemic ranged from 27.5% to 48% across different universities [10,11,12,13,14]. Compared to domestic students, international students face greater vulnerability to food insecurity, with estimates suggesting that up to 50% are affected [10,13].

This issue may be exacerbated due to the ongoing financial impact of COVID-19 and uncertain economic stability, resulting in high inflation rates [15]. Moreover, university students frequently encounter financial hardship due to rising tuition costs, high cost of housing and limited availability of affordable and nutritious food options on campus [16]. In addition, unequal access to food is increasingly evident across many Australian university campuses, largely due to the relative absence of subsidised food services and the high cost of healthier options [17,18]. The predominance of a market-oriented approach to food provision has further exacerbated the issue, making nutritious food less accessible [17]. A growing body of literature suggests that food insecurity is associated with an increased likelihood of poorer academic outcomes and adverse health consequences such as depression, poor sleep quality, and obesity [19]. The evidence implies that the significance of food insecurity extends far beyond hunger and inadequate diet quality and poses an urgent threat to academic success and to the physical and mental health of university students [20,21].

Contemporary university students in Australia represent an increasingly diverse population, including a growing proportion of mature-age students, individuals from migrant and low socioeconomic status backgrounds [22,23,24] and the number of international student enrolments in Australian higher education reached a new high in 2025 [25]. These demographic shifts have important implications for addressing food insecurity within higher education settings. Understanding the sociodemographic characteristics of students affected by food insecurity is therefore essential for developing targeted and effective interventions. Moreover, there is a notable lack of regular monitoring of food insecurity within Australian higher education settings [26,27]. To address this gap, this study aimed to assess the observed prevalence of food insecurity among Australian university students during the COVID-19 and post-COVID-19 and identify its sociodemographic associations with a view to targeting interventions for these populations.

## 2. Materials and Methods

### 2.1. Design and Participants

A cross-sectional survey was conducted biennially between 2020 and 2024 to measure food insecurity and other relevant factors between domestic and international students in Macquarie University, Sydney, Australia. All students aged at least 18 years and who were enrolled were eligible to participate in the survey. The university hosted a student population of over 40,000 students from 115 countries in 2024, encompassing a wide range of cultural backgrounds and socioeconomic status [28]. A convenience sample was used to recruit participants via social media, flyers on campus, digital displays and the university newsletter.

The recruitment advertisement consisted of a survey link and/or a quick response (QR) code, which directed interested participants to the Digital Participant Information and Consent Form (PICF). Consent was obtained prior to commencing the survey. Participation in the research was voluntary and anonymous. The first survey (Qualtrics, Provo, UT, USA, 2020) was conducted between 16 July 2020 and 20 September 2020, approximately 4 months after COVID restrictions were introduced and students moved to online learning. In the post-COVID period, the second wave survey was administered from October 2022 to January 2023, and the third wave survey was conducted from February to June 2024 (Figure S1, Supplementary Material), both via REDCap with CAPTCHA enabled.

During the second and third surveys, the research team also approached students on campus to participate in intercept surveys. Depending on their availability during the intercept surveys, students could opt to scan the QR code and complete the survey at their convenience or complete it immediately. The QR code was the primary response channel in both surveys. The online questionnaires and intercept survey were both identical. To enhance the response rate, participants who completed each survey were eligible to enter a lucky draw to win one of four gift vouchers valued at AUD 50.

#### Power Calculation

An a priori power calculation was not performed in the first survey because it was an exploratory survey conducted during the early stages of the COVID-19 pandemic. For the second survey, the target sample size was 160 domestic and 69 international students using a power calculation for proportions (https://sample-size.net/sample-size-proportions/, 20 September 2026). The sample size was calculated to achieve 80% power at α = 0.05 based on the assumption that the prevalence of food insecurity between domestic and international students would be differ by 20% as a conservative effect size based on the previous study [29], and the proportion of international students in the total sample would be 30%. For the third survey, the target sample size was 557 with 390 domestic and 167 international students using a power calculation for proportions (https://sample-size.net/sample-size-proportions/, 20 September 2026). The sample size was calculated to achieve 80% power at α = 0.05 based on the prevalence of food insecurity of domestic students being 13.0% with an odds ratio of 2.02 based on the assumption that the proportion of international students in the total sample would be 30% [14].

### 2.2. Measurements

#### 2.2.1. Food Insecurity

Student food insecurity status was measured using the six item Food Security Survey Module (FSSM), which assessed students’ food insecurity status over the past 12 months [30]. Food insecurity status was classified high food security (score 0), marginal food security (score 1), low food security (scores 2–4), or very low food security (scores 5–6).

#### 2.2.2. Sociodemographic Data

A range of sociodemographic characteristics including gender (female, male, other), age (<21 years, 21–25 years, 26–30 years, >30 years), student status (domestic/international), degree, employment status (full time, part time, casual, unemployed), household size, and location of residence (on-campus/residential postcode) were reported by participants. Based on the reported residential postcode, Socio-Economic Indicators for Areas (SEIFA) derived from the Index of Relative Socio-economic Disadvantage (IRSD) at Statistical Area Level 1 (SA1) were applied to determine the relative level of disadvantage [31] and for analytical purposes grouped in quintiles [32].

### 2.3. Data Analysis

Datasets were exported and cleaned using Stata for Windows, versions 18 and 19 (StataCorp, LP, College Station, TX, USA). Due to a very small sample of ‘other’ in gender, the analyses in this study excluded them (n = 21). Participants who did not complete the 6-item FSSM and participants who had missing values for key sociodemographic variables were excluded from the analysis (see Supplementary Figure S1). A score of 1 was given for each affirmative response to the 6-item FSSM. The total scores (0–6) were used to dichotomise the classification into food secure (0–1) and food insecure students (2–6). The prevalences of food security status were presented across the surveys and the pooled sample.

Descriptive analyses were used to compare the differences in sociodemographic variables across the surveys using chi-square tests for categorical variables. Pairwise tests (prtest) were conducted to compare differences in the prevalence of food insecurity as well as very low food security by survey year. Univariate logistic analyses were performed individually for each sociodemographic variable to estimate the unadjusted associations. Multivariable logistic regression models were performed to estimate adjusted odd ratios for food insecurity, controlling for sociodemographic variables (age, gender, degree, employment status, SEIFA, household size) and the survey year, further stratified by student status. Identified sociodemographic variables were included in the models based on the previous literature [33]. Additionally, a separate logistic regression analysis was performed to identify sociodemographic predictors of students who reported to have very low food security (scores 5–6), incorporating an interaction term between student status and survey year. Adjusted Odd Ratios (AORs) with corresponding CIs were reported. Collinearity in the multivariable model building was assessed using the Variance Inflation Factor (VIF) with a scores > 5 indicating issues with collinearity. The Pearson χ^2^ goodness-of-fit test (estat gof) was run to determine if the logistic models were a good fit and estimated predictive margins were estimated using the margins command in Stata to generate margins plots based on age groups and student status. Model diagnostics indicated satisfactory fit of the logistic regression models, and multicollinearity was low (mean VIF < 5). We also conducted a sensitivity analysis using the single-item measure of food insecurity used in the Australian National Health Survey [34], which adopted the item 1 “The food that I bought just didn’t last, and I didn’t have money to get more.” An affirmative response indicates food insecurity. This offers important context regarding the comparability of participants identified using different measurement approaches.

## 3. Results

The total sample of university students included in this study was 1010 (n = 103 for survey 2020, n = 232 for survey 2022, n = 675 for survey 2024). Table 1 shows that most participants were female, undergraduate students, employed casually, and living in the least disadvantaged areas (SEIFA 5). There were significant differences in employment and SEIFA by student status (see Supplementary Table S1). International students were more likely to be unemployed (38.3%) than domestic students (19.9%), whereas domestic students were more likely to be employed casually (46.4% vs. 27.3%). Compared with domestic students, international students were more likely to reside in the most disadvantaged areas (12.3% vs. 8.2%). Most students in both groups resided in the least disadvantaged areas, although this proportion was higher among international students (76.0% vs. 70.6%).

### 3.1. Food Insecurity Status Across Surveys

Our surveys indicate that the prevalence of very low food security worsened, rising from 7.8% in 2020 to 16.3% in 2024 (Table 2). An independent-samples proportions test showed a significant increase (*p* = 0.025) (Supplementary Table S2). Based on student status (Figure 1), the prevalence of food insecurity fluctuated across surveys. However, the overall prevalence among university students has remained relatively consistent at around 40% since 2020. One encouraging finding was the improvement in the prevalence of high food security from 1.9% in 2020 to 49.9% in 2024. A chi-square test showed no significant association between survey year and food insecurity status, χ^2^(2) = 0.12, *p* = 0.942. Further results from pairwise proportion tests of food insecurity status between survey years are available in Supplementary Table S3.

### 3.2. Association Between Food Insecurity and Sociodemographic Variables

Table 3 presents the results of multivariable logistic regression analyses and further stratified by student status. International students had significantly higher odds of being food insecure compared to domestic students after adjusting for sociodemographic and survey year (AOR 2.34, 95% CI 1.80–3.04, *p* < 0.001). Compared with students younger than 21 years old, those aged 21–25 years (AOR 1.65, 95% CI 1.20, 2.20, *p* = 0.002), 26–30 years (AOR 2.00, 95% CI 1.36, 2.94, *p* < 0.001) and >30 years (AOR 1.67, 95% CI 1.11, 2.52, *p* = 0.014 were associated with significantly higher odds of experiencing food insecurity in the pooled analysis. Model diagnostics indicated satisfactory fit of the logistic regression model. Multicollinearity was low (mean VIF = 2.16).

In separate interaction analyses, a significant interaction was observed between student status and survey year, indicating that the excess odds of food insecurity among international students varied across survey years (see Supplementary Table S4). No significant interaction was observed between gender and survey year (see Supplementary Table S5). Adjusted predicted probabilities indicated that postgraduate students were more likely to experience food insecurity than undergraduates in 2020, whereas undergraduates had higher probabilities of food insecurity in 2022 and 2024 (see Figure 2, Supplementary Table S6).

### 3.3. International vs. Domestic Students

Our surveys showed that there was a significant difference in some characteristics between international and domestic students, including age group, education degrees, employment status, SEIFA, and household size (see Supplementary Table S1). Student status was significantly associated with employment status (χ^2^(3) = 56.88, *p* < 0.001), with domestic students more likely to be casually employed (46.4% vs. 27.3%) and international students more likely to be unemployed (38.3% vs. 19.9%). Student status was further associated with SEIFA quintile (χ^2^(4) = 20.00, *p* = 0.001), with international students disproportionately represented in more disadvantaged areas.

Stratified analyses by student status showed that changes in food insecurity across survey years did not differ by degree levels among either domestic or international students (Table 4). Household size was associated with food insecurity, but patterns differed by student status. Compared with living alone, living in a 4–5 person household was associated with lower odds of food insecurity among domestic students (AOR = 0.29, 95% CI: 0.13–0.66, *p* = 0.003), whereas larger households were associated with higher odds among international students (≥6 people: AOR = 2.40, 95% CI: 1.00–5.76, *p* = 0.049) (Table 4).

### 3.4. Sociodemographic Predictors of Very Low Food Security

International students had higher odds of very low food security than domestic students (AOR = 7.00, 95% CI: 1.27–38.58, *p* = 0.025), although this difference was significantly attenuated in 2022 (interaction AOR = 0.13, 95% CI: 0.02–0.89, *p* = 0.038) (Table 5). As shown in Supplementary Figure S3, adjusted predicted probabilities of very low food security were generally higher among international students across survey years, with the smallest difference observed in 2022. In Table 5, postgraduate students had lower odds of very low food security than undergraduates (OR = 0.42, 95% CI: 0.25–0.73, *p* = 0.002), whereas students aged 21–25 years had higher odds than the youngest age group (OR = 1.69, 95% CI: 1.03–2.77, *p* = 0.039). Multicollinearity was low (mean VIF = 3.24).

### 3.5. Sensitivity Analysis

In the sensitivity analysis using the single item measure, the association between student status and food insecurity was consistently significant after adjusting for covariates. International students exhibited higher adjusted odds of experiencing food insecurity compared to domestic students. Additionally, certain age groups (students aged 26–30 years and >30 years) and postgraduate also remained significantly associated with food insecurity. In contrast, males were significantly more likely to be food insecure than females. (see Supplementary Table S7).

## 4. Discussion

This study provides compelling evidence that food insecurity among university students is not a transient consequence of the COVID-19 pandemic but a persistent and potentially worsening challenge within the higher education setting. Our study highlights that food insecurity among Australian university students in a metropolitan area is unacceptably high and remained persistently around 40% over this period. This figure is similar to the pooled estimate (of 42.2%) for food insecurity among post-secondary students in high income countries [19]. The prevalence of very low food security in this study increased from 7.8% in 2020 to 16.3% in 2024, indicating a concerning upward trend. Our findings further corroborate evidence from a repeated cross-sectional study at another Australian university in Tasmania [27]. This underscores the severity of this issue and its potential long-term implications. International students are consistently identified as being at a higher risk of experiencing food insecurity compared to domestic students, highlighting the importance of addressing underlying socioeconomic barriers that disproportionally affect international students.

### 4.1. Social Determinants of Health

Although a recent scoping review highlights a substantial increase in studies on campus-based programs addressing food insecurity among higher education settings between 2020 and 2023 [35], multiple studies continue to report persistently high levels of food insecurity among university students in high income countries [19]. A recent scoping review using the WHO Commission on the Social Determinants of Health Framework highlights how structural (e.g., student status) and intermediary determinants (e.g., food insecurity, housing issues, social isolation) affect the experiences and wellbeing of international students compared with domestic students [36].

Pathways to food insecurity are complex and often associated with other social determinants of health, such as housing. A growing body of literature suggests that housing instability has a negative association with food insecurity [37,38,39]. Unlike in the US, university-sponsored accommodation is not common in Australia, requiring the majority of students living away from home to secure housing on the open rental market [40]. In particular, the current Australia’s housing crisis has worsened food insecurity in general. The Australian Institute of Family Studies reported that an increasing proportion of young adults continue to live in the parental home well into their late adolescents and early twenties, with 72% of 19 years olds living with their parents in 2021 [41]. This may suggest that university students are leaving the parental home at later ages, and this transition can have substantial implications for their financial stability.

Our stratified analysis by student status showed that domestic students living in households of 4–5 members had lower odds of food insecurity compared with those living alone. We presume these numbers reflect the average size of a family household, suggesting that students in this category are likely to still be living with their family in the parental home. On the other hand, the odds of food insecurity were higher among international students living in a larger household size (at least six household members). For many international students, rental affordability is a major concern even before the COVID-19 pandemic [42]. A report on housing, work, study, and wellbeing among international students highlighted that only 36% of students agreed that it was easy to afford their housing costs in 2019. The difficulties in paying rent rose sharply during the COVID-19 pandemic in 2020 with 54% reporting that they found themselves worrying about paying the rent each week. A documentary analysis on international student housing shows that existing policy and regulatory instruments in Australia do not mandate the provision of affordable or safe accommodation for international students, leaving their vulnerability inadequately addressed [43].

Our study found that employment status and SEIFA were not significantly associated with food insecurity, which is similar to the findings from another study in food security and socioeconomic disadvantage in Australia [44]. A possible explanation for the lack of variance in levels of food insecurity across different sociodemographic groups is the implementation of government policies related to financial support (e.g., income assistance, unlimited working hours), which were accessible to recipients who met the eligibility criteria during this period. For domestic students specifically, those who are unemployed and in more disadvantaged areas are more likely to be eligible for student income support payments [45]. Moreover, the majority of university students in this study were either engaged in casual employment or were unemployed, whereas a relatively smaller proportion held full-time or part-time positions. Employment status alone may not adequately capture students’ financial capacity, as it does not account for important factors such as income level, hours worked, or employment stability. It is worth noting that distribution of living location differed between international and domestic students in our sample, with international students disproportionately represented in more disadvantaged areas. Further investigation is warranted to better understand how this may contribute to differences in food insecurity between the two groups.

While it is encouraging that the prevalence of high food security increased from 1.9% in 2020 to 49.9% in 2024; the prevalence of very low food security also increased from 7.8% to 16.3% in the same period. This pattern suggests a polarisation of students’ experiences and financial circumstances. Our finding that the prevalence of food insecurity among domestic students nearly doubled between 2020 and 2022, while its prevalence among international students declined significantly during the same period, aligns with the timing of policy differences between these two groups. In 2020, the Australian government introduced COVID-19 financial support programs for citizens and permanent residents, but temporary residents, such as international students were ineligible [46]. These programs were discontinued in early 2022, which may have contributed to increased financial stress in the post-pandemic period for those who had previously relied on such assistance. A similar timeframe was observed for the increase in food insecurity prevalence among domestic students. In comparison, the government removed all work restrictions for all student visa holders in early 2022 and this policy ended on 30 June 2023 [47]. During the relaxation of student work visas, international students were permitted to work full-time, thereby this period coincided with a decline in food insecurity prevalence among international students. However, the prevalence of food insecurity rose again in 2024 once work-hour limits were re-introduced. These trends can be contextualised within the broader landscape of Australian income-support and visa-work policy during this period. Although we acknowledge that this study did not collect data on whether the students received the financial support or their actual working hours, government policies remain a relevant contextual factor for understanding food security among university students. This underscores the need for Australian law and policy in food security to effectively address and mitigate the long-term risks associated with food insecurity.

Additionally, our sensitivity analysis indicated that the associations observed for some covariates, particularly gender and household size, varied according to the definition of food insecurity used. In contrast, international student status remained a significant predictor across all model specifications, demonstrating the robustness of this association regardless of how food insecurity was operationalised. These findings highlight the methodological implications of food insecurity measurement, as the significance of some covariates varied according to the operational definition used. A systematic review of measuring and understanding food insecurity in Australia highlighted the need for greater consistency in the measurement of food insecurity and for the development of food insecurity instruments tailored for the Australian context [48].

### 4.2. Strengths and Limitations

To our knowledge, this study is one of a very few studies to include multiple survey waves assessing food security status among university students in Australia from 2020 to 2024. The strengths of our study include the collection of data on the social determinants of health that are related with food insecurity such as student visa status. We also used a validated measure, the 6-item USDA FSSM for analysis to ensure consistency in measuring food security status across surveys; however, we recognise that this tool may underestimate the prevalence and severity of food insecurity compared to more comprehensive versions of the FSSM (i.e., 18-item Household FSSM) [34]. Despite this limitation, the 6-item module has the advantage of a lower respondent burden and offers a high accuracy.

Several variables that may act as confounders (e.g., psychological distress, income, housing) were not consistently collected across surveys. As the questionnaire was iteratively updated across waves to incorporate emerging priorities relevant to each survey period, some variables were added or removed over time, and comprehensiveness was balanced against the need to keep the instrument concise and minimise respondent burden. Although several associations in the current study were statistically significant, the wide confidence intervals observed for some estimates suggest limited precision. Therefore, the findings should be interpreted with caution, and further research with larger samples is needed to confirm these associations.

It is worth nothing that recruitment strategy changed across surveys. The first survey used online advertised exclusively, whereas the second and third surveys supplemented this advertisement with the intercept survey and in-person QR-code recruitment. As some sample characteristics varied significantly between surveys, the findings should be interpreted cautiously. The cross-sectional design limits our ability to draw causal inferences about the associations between food security and socioeconomic predictors in this study. Other limitations include that all surveys relied on convenience sampling and self-reported data, which may have introduced selection bias and been subject to social desirability and recall bias. Furthermore, all surveys were conducted within a single university in a metropolitan area using convenience sampling, which limits generalisability to the broader Australian tertiary sector [34], as drivers of food insecurity may differ substantially in outer-metropolitan and regional settings [18]. Future research could consider collecting data on the broader structural factors, including whether students received financial support from the university or government. Incorporating qualitative data would also help explain the underlying reasons for food insecurity and provide a better understanding of housing and financial factors impacting university students’ food security status.

## 5. Conclusions

Our repeated cross-sectional study suggests that food insecurity remains a critical and ongoing public health issue within higher education settings. Notably, the proportion of students experiencing very low food security increased the most, with international students and certain age groups disproportionately affected. Our findings also suggest that household size may play a more complex and previously underexplored role in food security than has been recognised. Further studies using larger samples and more representative samples is warranted. Ongoing monitoring and reporting of food insecurity and its associations with social determinants is needed to inform targeted interventions and policy responses within higher education settings and government levels.

## Figures and Tables

**Figure 1:**
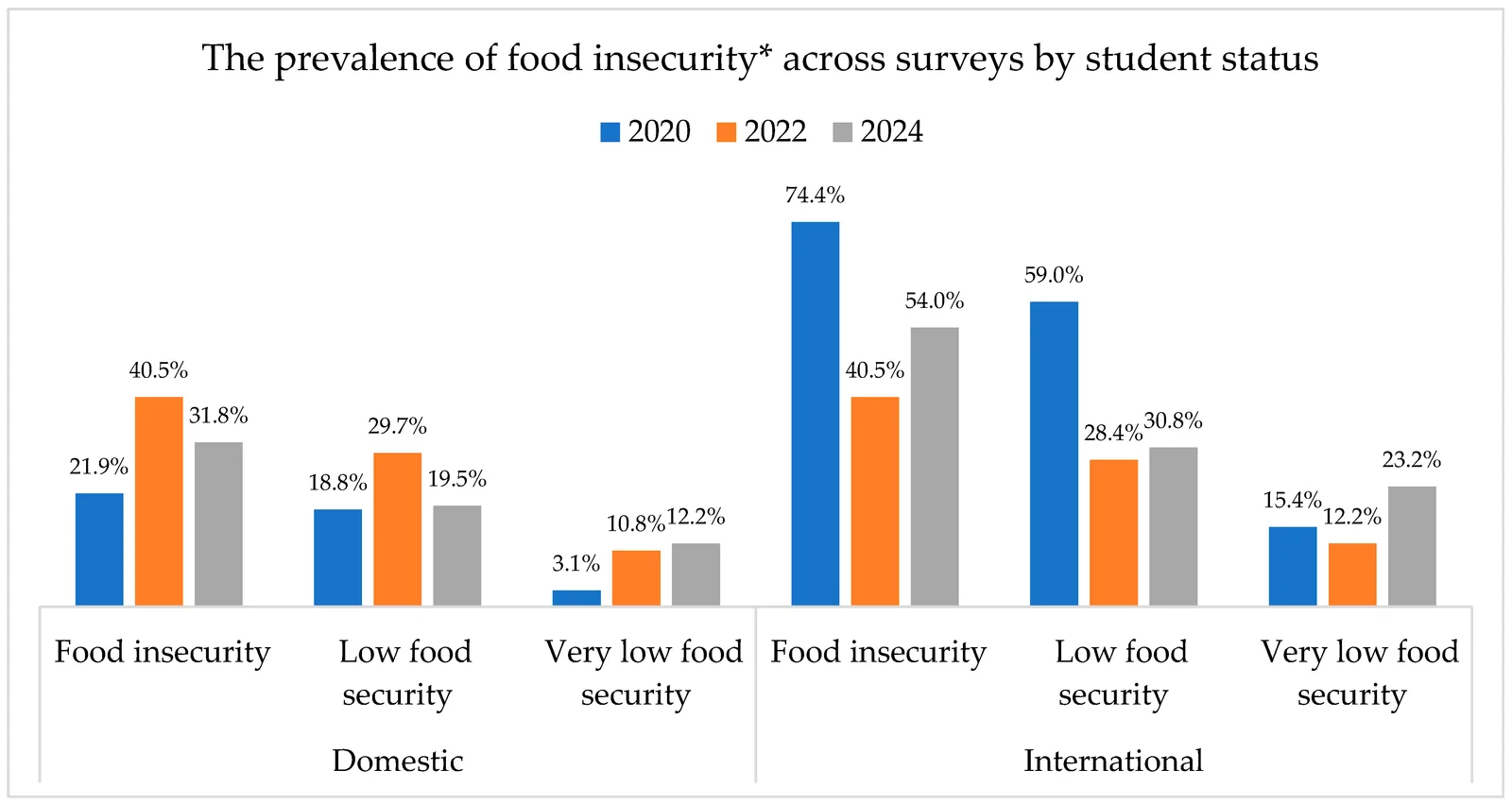
The prevalence of food insecurity among university students based on student status (domestic vs. international). * Low food security and very low food security are classified as food insecurity.

**Figure 2:**
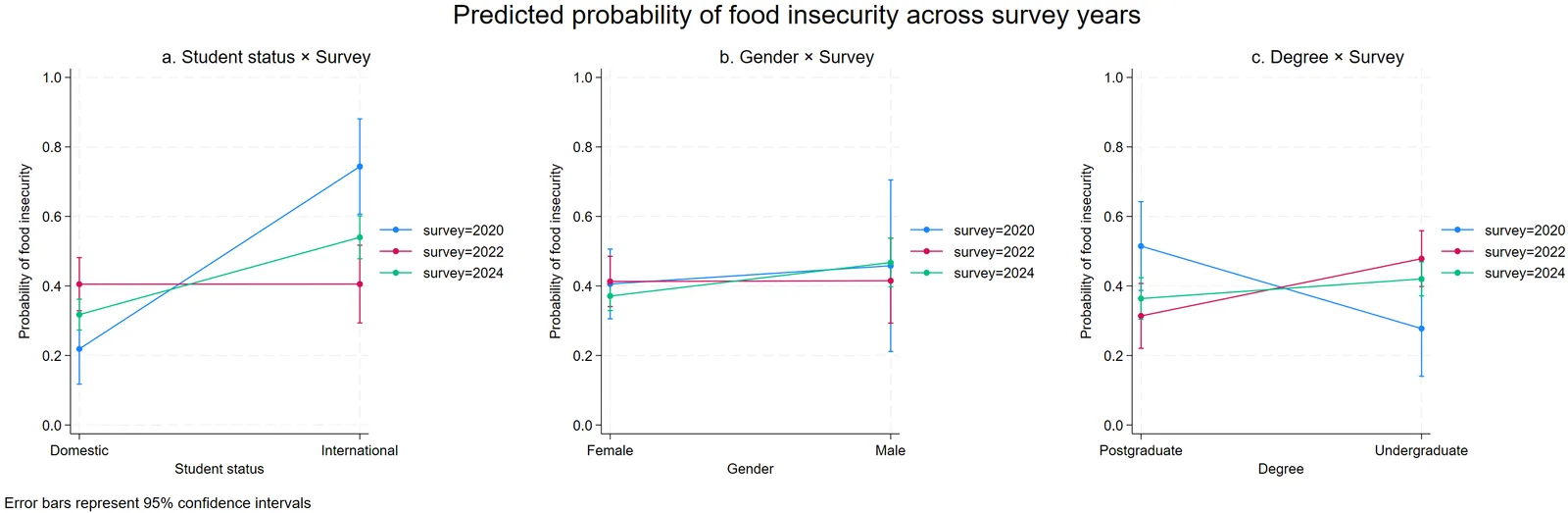
Adjusted predicted probability of food insecurity by (**a**) student status, (**b**) gender, and (**c**) degree level, across survey years. Predicted probabilities are marginal estimates from three separate logistic regression models, each including an interaction between the demographic variable of interest and survey year; models for gender and degree level were additionally adjusted for student status.

**Table 1:** Sociodemographic characteristics of the study sample at each survey and overall sample.

	2020	2022	2024	All	
	n = 103	n = 232	n = 675	n = 1010	
Variable	n (%)	n (%)	n (%)	n (%)	*p*-Value
**Age group**					
<21 years	28 (27.7%)	85 (36.8%)	257 (38.1%)	370 (36.7%)	0.232
21–25 years	35 (34.7%)	81 (35.1%)	238 (35.3%)	354 (35.2%)	
26–30 years	24 (23.8%)	34 (14.7%)	96 (14.2%)	154 (15.3%)	
>30 years	14 (13.9%)	31 (13.4%)	84 (12.4%)	129 (12.8%)	
**Gender**					
Female	88 (85.4%)	172 (74.1%)	488 (72.3%)	748 (74.1%)	0.018
Male	15 (14.6%)	60 (25.9%)	187 (27.7%)	262 (25.9%)	
**Student status**					
Domestic	64 (62.1%)	158 (68.1%)	425 (63.0%)	647 (64.1%)	0.339
International	39 (37.9%)	74 (31.9%)	250 (37.0%)	363 (35.9%)	
**Degree**					
Postgraduate	58 (56.9%)	86 (37.1%)	259 (38.4%)	403 (39.9%)	0.001
Undergraduate	44 (43.1%)	146 (62.9%)	416 (61.6%)	606 (60.1%)	
**Employment status**					
Full time	4 (3.9%)	38 (16.4%)	60 (8.9%)	102 (10.1%)	<0.001
Part time	14 (13.6%)	61 (26.3%)	166 (24.6%)	241 (23.9%)	
Casual	41 (39.8%)	100 (43.1%)	258 (38.2%)	399 (39.5%)	
Unemployed	44 (42.7%)	33 (14.2%)	191 (28.3%)	268 (26.5%)	
**SEIFA (IRSD)**					
1 (most disadvantaged)	13 (12.9%)	10 (4.4%)	73 (10.9%)	96 (9.7%)	0.002
2	7 (6.9%)	3 (1.3%)	15 (2.2%)	25 (2.5%)	
3	9 (8.9%)	19 (8.4%)	62 (9.3%)	90 (9.1%)	
4	10 (9.9%)	13 (5.8%)	39 (5.8%)	62 (6.2%)	
5 (least disadvantaged)	62 (61.4%)	180 (80.0%)	479 (71.7%)	721 (72.5%)	
**Household size**					
Alone	5 (4.9%)	25 (11.0%)	49 (7.3%)	79 (7.9%)	0.060
2–3	46 (45.1%)	83 (36.6%)	224 (33.2%)	353 (35.2%)	
4–5	40 (39.2%)	87 (38.3%)	316 (46.8%)	443 (44.1%)	
6 or more	11 (10.8%)	32 (14.1%)	86 (12.7%)	129 (12.8%)	

**Table 2:** Food insecurity classification at each survey and the pooled sample.

Food Security Status	2020 [95% CI]	2022 [95% CI]	2024 [95% CI]	All [95% CI]
**Food security**	58.3% [48.5–67.4]	59.5% [53.0–65.6]	60.0% [56.2–63.6]	59.7% [56.6–62.7]
**High food security**	1.9% [0.5–7.4]	43.5% [37.3–50.0]	49.9% [46.2–53.7]	43.6% [40.5–46.6]
**Marginal food security**	56.3% [46.6–65.6]	15.9% [11.8–21.2]	10.1% [8.0–12.6]	16.1% [14.0–18.5]
**Food insecurity**	41.7% [32.6–51.5]	40.5% [34.4–47.0]	40.0% [36.4–43.8]	40.3% [37.3–43.4]
**Low food security**	34.0% [25.5–43.6]	29.3% [23.8–35.5]	23.7% [20.6–27.1]	26.0% [23.4–28.8]
**Very low food security**	7.8% [3.9–14.8]	11.2% [7.7–16.0]	16.3% [13.7–19.3]	14.3% [12.2–16.6]

**Table 3:** Univariate and multivariable logistic regression analyses of factors associated with food insecurity among university students.

Food Security Status ^a^		Univariate			Multivariable ^b^		
Variable	Category	OR	95% CI	*p*-Value	AOR	95% CI	*p*-Value
**Student status**	Domestic	REF					
	International	2.34	1.80–3.04	<0.001	2.72	1.93–3.84	<0.001
**Age group**	<21 years	REF					
	21–25 years	1.62	1.20–2.20	0.002	1.6	1.12–2.29	0.010
	26–30 years	2	1.36–2.94	<0.001	1.78	1.06–2.98	0.028
	>30 years	1.67	1.11–2.52	0.014	1.88	1.09–3.24	0.023
**Gender**	Female	REF					
	Male	1.36	1.02–1.80	0.035	1.33	0.98–1.79	0.067
**Degree**	Postgraduate	1.35	1.04–1.74	0.022	0.57	0.38–0.85	0.006
	Undergraduate	REF					
**Survey year**	2020	REF					
	2022	0.95	0.59–1.52	0.833	0.79	0.47–1.33	0.377
	2024	0.93	0.61–1.42	0.736	0.84	0.53–1.33	0.451
**Employment status**	Full time	REF					
	Part time	1.1	0.69–1.75	0.689	1.31	0.80–2.16	0.287
	Casual	0.69	0.45–1.08	0.102	0.86	0.53–1.40	0.545
	Unemployed	0.87	0.55–1.38	0.548	0.84	0.50–1.39	0.485
**SEIFA (IRSD)**	1 (most disadvantages)	0.96	0.62–1.49	0.864	0.85	0.54–1.35	0.497
	2	0.98	0.43–2.21	0.960	1.25	0.52–2.96	0.620
	3	1.03	0.66–1.60	0.911	1.31	0.82–2.10	0.252
	4	0.93	0.54–1.58	0.783	1.05	0.60–1.86	0.854
	5 (least disadvantages)	REF					
**Household size**	Alone	REF					
	2–3	0.91	0.56–1.48	0.699	1.1	0.66–1.86	0.710
	4–5	0.68	0.42–1.10	0.114	0.86	0.51–1.45	0.567
	6 or more	1.24	0.71–2.17	0.457	1.46	0.80–2.67	0.217

^a^ Food security status (total scores of 2–6); ^b^ Adjusted for sociodemographic variables: gender, age groups, education degrees, survey year, employment status, SEIFA, household size. AOR, adjusted odds ratio; CI, confidence of interval; OR, Odds ratio; SEIFA, socio-economic indexes for areas. SEIFA based on index of relative socio-economic disadvantage (IRSD): Q1 the most disadvantages and Q5 the least disadvantages.

**Table 4:** Multivariable logistic regression analyses of factors associated with food insecurity by student status.

		International Students ^b^			Domestic Students ^b^		
Food Security Status ^a^		AOR	95% CI	*p*-Value	AOR	95% CI	*p*-Value
**Age group**	<21 years	REF					
	21–25 years	0.59	0.26–1.37	0.222	1.82	1.20–2.74	0.005
	26–30 years	0.70	0.28–1.75	0.442	2.00	0.88–4.54	0.096
	>30 years	1.14	0.41–3.16	0.808	1.15	0.53–2.51	0.730
**Gender**	Female						
	Male	1.42	0.86–2.36	0.172	1.27	0.85–1.89	0.237
**Degree**	Postgraduate	1.10	0.55–2.18	0.785	0.44	0.25–0.76	0.004
	Undergraduate	REF					
**Survey year**	2020	REF					
	2022	0.13	0.05–0.36	<0.001	2.24	1.05–4.75	0.036
	2024	0.27	0.11–0.65	0.003	1.71	0.85–3.45	0.132
**Employment status**	Full time	REF					
	Part time	1.54	0.71–3.34	0.273	1.00	0.51–1.99	0.994
	Casual	1.02	0.48–2.20	0.951	0.64	0.33–1.24	0.184
	Unemployed	0.79	0.37–1.66	0.532	0.60	0.29–1.25	0.171
**SEIFA**	1	0.68	0.34–1.36	0.275	0.76	0.39–1.52	0.442
	2	0.54	0.13–2.18	0.388	1.84	0.57–5.86	0.305
	3	1.42	0.51–3.98	0.507	1.23	0.71–2.15	0.462
	4	0.53	0.15–1.86	0.318	1.19	0.62–2.32	0.600
	5	REF					
**Household size**	Alone	REF					
	2–3	1.26	0.58–2.72	0.558	0.62	0.28–1.36	0.234
	4–5	2.10	0.99–4.47	0.055	0.29	0.13–0.66	0.003
	6 or more	2.40	1.00–5.76	0.049	0.61	0.24–1.56	0.306

^a^ Food security status (total scores of 2–6); ^b^ Adjusted for sociodemographic variables: age groups, gender, education degrees, survey year, employment status, SEIFA, household size. AOR, adjusted odds ratio; CI, confidence of interval; SEIFA, socio-economic indexes for areas. SEIFA based on index of relative socio-economic disadvantage (IRSD): Q1 the most disadvantages and Q5 the least disadvantages.

**Table 5:** Logistic regression analysis of factors associated with very low food security among university students.

Very Low Food Security ^a^		Multivariable ^b^		
Variables	Category	AOR	95% CI	*p*-Value
**Student status**	Domestic	REF		
	International	7.00	1.27–38.58	0.025
**Survey year**	2020	REF		
	2022	3.36	0.73–15.37	0.118
	2024	3.79	0.88–16.28	0.073
**Student status × survey year interaction**	International × 2020	REF		
	International × 2022	0.13	0.02–0.89	0.038
	International × 2024	0.38	0.07–2.16	0.275
**Gender**	Female	REF		
	Male	1.47	0.98–2.19	0.061
**Age group**	<21 years	REF		
	21–25 years	1.69	1.03–2.77	0.039
	26–30 years	1.77	0.88–3.56	0.110
	>30 years	2.06	0.99–4.26	0.052
**Degree**	Undergraduate	REF		
	Postgraduate	0.42	0.25–0.73	0.002
**Employment status**	Full time	REF		
	Part time	0.84	0.44–1.59	0.588
	Casual	0.60	0.32–1.13	0.114
	Unemployed	0.70	0.36–1.34	0.281
**SEIFA (IRSD)**	5 (least disadvantages)			
	4	1.49	0.70–3.19	0.301
	3	1.28	0.65–2.50	0.476
	2	2.71	0.92–7.96	0.069
	1 (most disadvantages)	1.17	0.63–2.14	0.623
**Household size**	Alone			
	2–3	0.64	0.34–1.21	0.172
	4–5	0.38	0.20–0.74	0.004
	6 or more	0.73	0.35–1.53	0.409

^a^ Very low food security referred to participants who had total scores of 5–6; ^b^ Adjusted for sociodemographic variables: gender, age groups, education degrees, survey year, employment status, SEIFA, household size. AOR, adjusted odds ratio; CI, confidence of interval; SEIFA, socio-economic indexes for areas. SEIFA based on index of relative socio-economic disadvantage (IRSD): Q1 the most disadvantages and Q5 the least disadvantages.

## Data Availability

The data presented in this study are available on request from the corresponding author. The data are not publicly available due to privacy or ethical restrictions.

## Supplementary Materials

The following supporting information can be downloaded at: https://www.mdpi.com/article/10.3390/nu18193124/s1, Figure S1: The flowchart of food insecurity surveys; Figure S2. Adjusted predicted probabilities of food insecurity by degree level and survey year among (A) international and (B) domestic students. Error bars represent 95% confidence intervals.; Figure S3. Adjusted predicted probabilities of very low food security by student status and survey year. Error bars represent 95% confidence intervals.; Table S1. Food insecurity status and sociodemographic characteristics based on student status; Table S2. Pairwise comparisons ofvery lowfood security status between survey years; Table S3. Pairwise comparisons of food insecurity status between survey years; Table S4. Logistic regression estimating adjusted odds of food insecurity by student status and survey wave, including a student status × survey year interaction term; Table S5. Logistic regression estimating adjusted odds of food insecurity by student status, gender, survey year, and gender × survey interaction; Table S6. Logistic regression estimating adjusted odds of food insecurity by student status, degree, survey year, and degree × survey interaction; Table S7. Sensitivity analysis of factors associated with food insecurity among university students.
