# Supplementary material for: Food Insecurity Among University Students: Evidence from Three Cross-Sectional Surveys, 2020–2024
Source: Nutrients. 2026 Sep 23;18(19):3124. doi: 10.3390/nu18193124 (PMC13666799; doi:10.3390/nu18193124)
Supplement: Supplementary file 1 [file nutrients-18-03124-s001.zip › nutrients-4498349-supplementary.pdf]

Supplementary materials

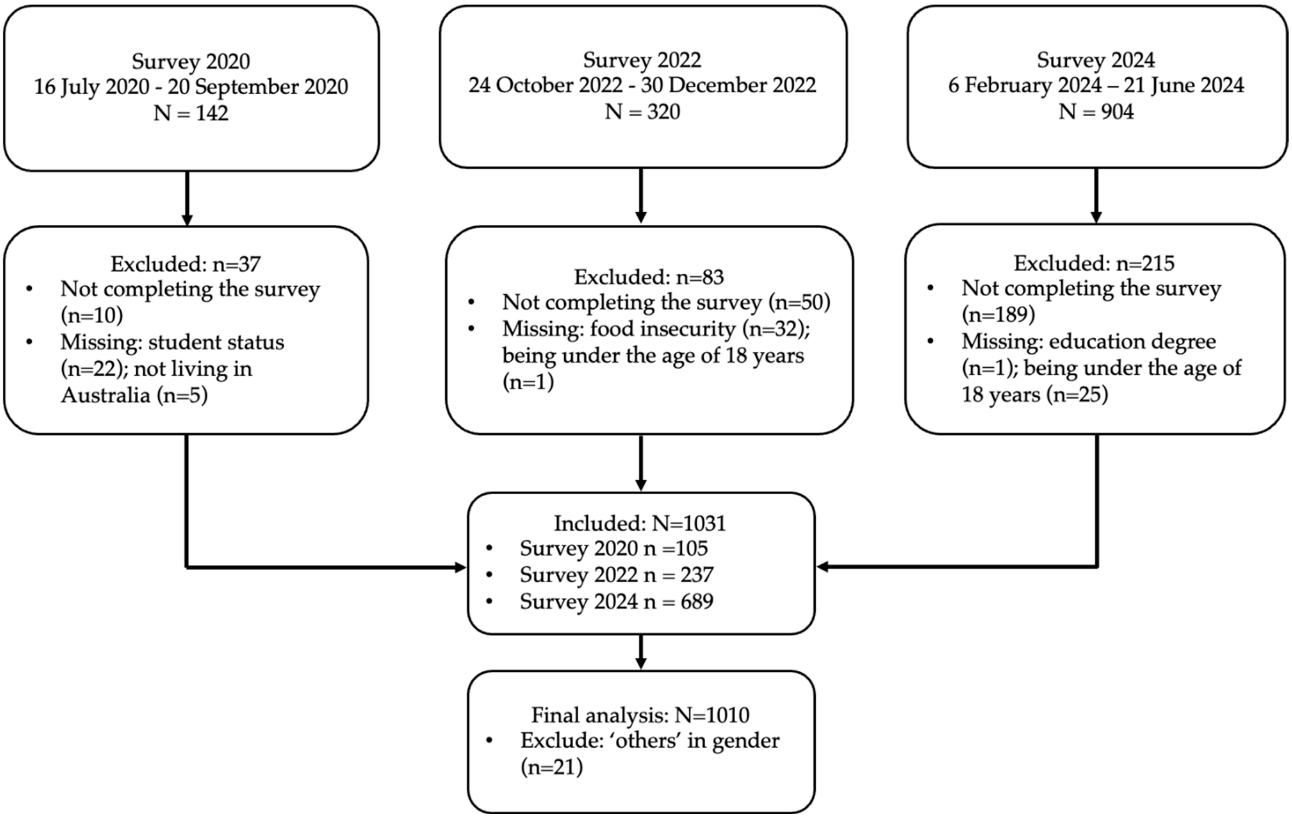

Figure S1. The flowchart of food insecurity surveys

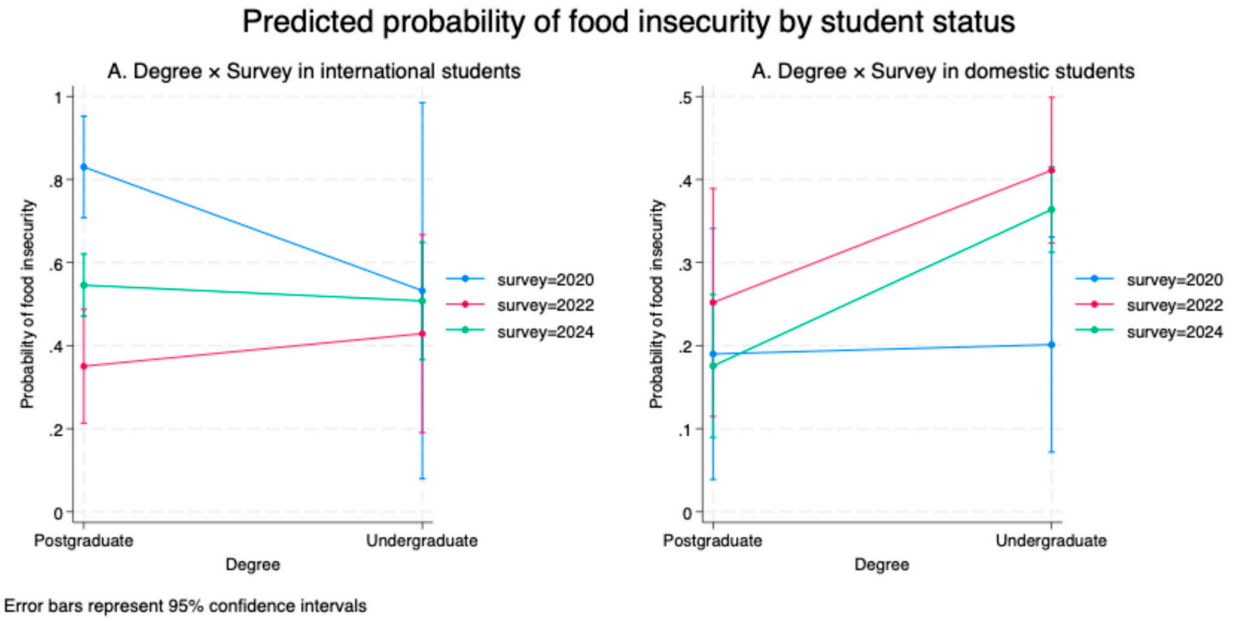

Figure S2. Adjusted predicted probabilities of food insecurity by degree level and survey year among (A) international and (B) domestic students. Error bars represent 95% confidence intervals.

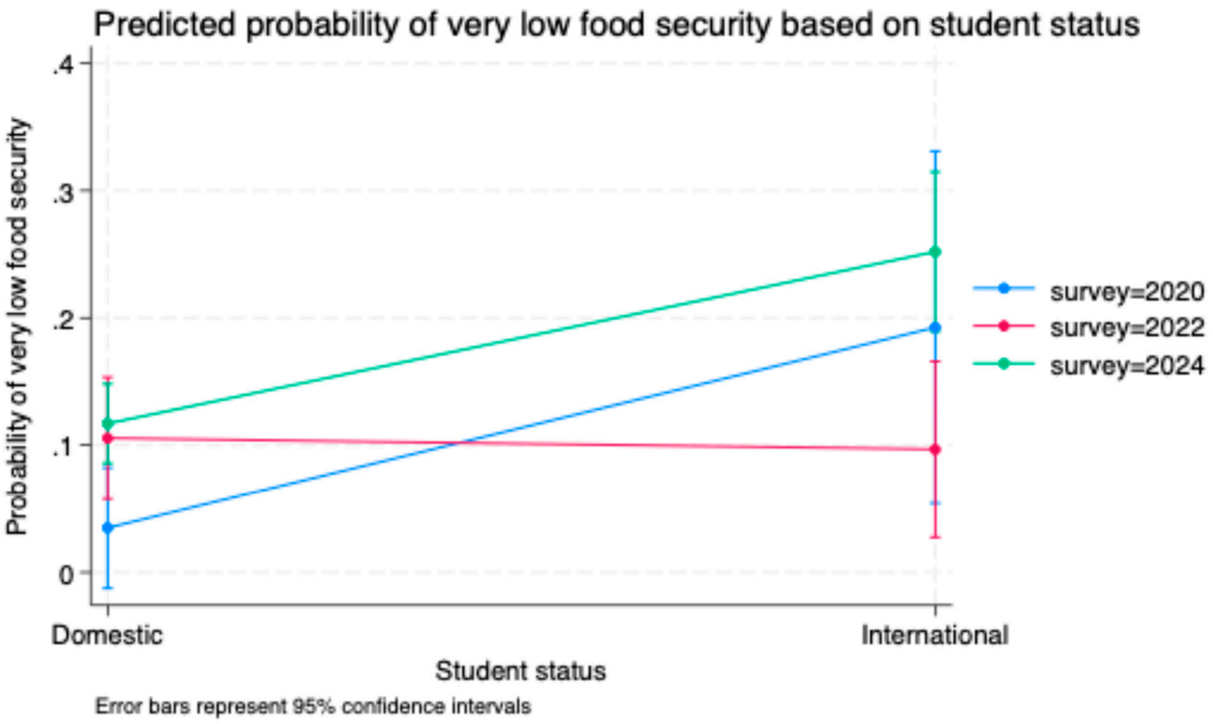

Figure S3. Adjusted predicted probabilities of very low food security by student status and survey year. Error bars represent 95% confidence intervals.

Supplementary Table S1. Food insecurity status and sociodemographic characteristics based on student status

	Domestic n (%)	International n (%)	p-value
	647 (64.1%)	363 (35.9%)	
Food security status			
Food security	434 (67.1%)	169 (46.6%)	<0.001
Food insecurity	213 (32.9%)	194 (53.4%)	
Survey year			
2020	64 (9.9%)	39 (10.7%)	0.339
2022	158 (24.4%)	74 (20.4%)	
2024	425 (65.7%)	250 (68.9%)	
Age group			
<21 years	318 (49.3%)	52 (14.4%)	<0.001
21-25 years	215 (33.3%)	139 (38.4%)	
26-30 years	44 (6.8%)	110 (30.4%)	
>30 years	68 (10.5%)	61 (16.9%)	
Gender			
Female	483 (74.7%)	265 (73.0%)	0.566
Male	164 (25.3%)	98 (27.0%)	
Degree			
Postgraduate	132 (20.4%)	271 (74.7%)	<0.001
Undergraduate	514 (79.6%)	92 (25.3%)	
Employment status			
Full time	55 (8.5%)	47 (12.9%)	<0.001
Part time	163 (25.2%)	78 (21.5%)	
Casual	300 (46.4%)	99 (27.3%)	
Unemployed	129 (19.9%)	139 (38.3%)	
SEIFA (IRSD)			
1 (most disadvantaged)	52 (8.2%)	44 (12.3%)	<0.001
2	15 (2.4%)	10 (2.8%)	
3	71 (11.2%)	19 (5.3%)	
4	49 (7.7%)	13 (3.6%)	
5 (least disadvantaged)	448 (70.6%)	273 (76.0%)	
Household size			
Alone	35 (5.4%)	44 (12.2%)	<0.001
2-3	227 (35.2%)	126 (35.0%)	
4-5	307 (47.7%)	136 (37.8%)	
6 or more	75 (11.6%)	54 (15.0%)	

Supplementary Table S2. Pairwise comparisons of very low food security status between survey years

Comparison	N1	Proportion 1	95% CI 1	N2	Proportion 2	95% CI 2	p-value
2020 vs 2022	103	7.8%	(2.60, 12.94)	232	11.2%	(7.15, 15.27)	0.336
2020 vs 2024	103	7.8%	(2.60, 12.94)	675	16.3%	(13.51, 19.08)	0.025
2022 vs 2024	232	11.2%	(7.15, 15.27)	675	16.3%	(13.51, 19.08)	0.061

Supplementary Table S3. Pairwise comparisons of food insecurity status between survey years

Comparison	N1	Proportion 1	95% CI 1	N2	Proportion 2	95% CI 2	p-value
2020 vs 2022	103	41.7%	(32.22, 51.27)	232	40.5%	(34.20, 46.83)	0.833
2020 vs 2024	103	41.7%	(32.22, 51.27)	675	40.0%	(36.30, 43.70)	0.736
2022 vs 2024	232	40.5%	(34.20, 46.83)	675	40.0%	(36.30, 43.70)	0.890

Supplementary Table S4. Logistic regression estimating adjusted odds of food insecurity by student status and survey wave, including a student status × survey year interaction term

		AOR	95% CI	p-value
Student status	Domestic	REF		
	International	10.36	4.08-26.29	<0.001
Survey year	2020	REF		
	2022	2.43	1.24-4.76	0.010
	2024	1.66	0.89-3.11	0.112
Interaction: student status x survey year	International x 2020	REF		
	International x 2022	0.10	0.03-0.29	0.000
	International x 2024	0.24	0.09-0.65	0.005

AOR, adjusted odds ratio; CI, confidence of interval; REF, reference group

Supplementary Table S5. Logistic regression estimating adjusted odds of food insecurity by student status, gender, survey year, and gender × survey interaction

		AOR	95% CI	p-value
Student status	Domestic	REF		
	International	2.35	1.80-3.07	<0.001
Gender	Female	REF		
	Male	1.25	0.41-3.84	0.698
Survey year	2020	REF		
	2022	1.03	0.60-1.76	0.910
	2024	0.86	0.53-1.38	0.526
Interaction: gender x survey year	Male x 2020	REF		
	Male x 2022	0.81	0.22-2.90	0.744
	Male x 2024	1.21	0.37-3.93	0.749

AOR, adjusted odds ratio; CI, confidence of interval; REF, reference group

Supplementary Table S6. Logistic regression estimating adjusted odds of food insecurity by student status, degree, survey year, and degree × survey interaction

		AOR	95% CI	p-value
Student status	Domestic	REF		
	International	2.69	1.95-3.70	<0.001
Degree	Postgraduate	2.92	1.19-7.20	0.020
	Undergraduate	REF		
Survey year	2020	REF		
	2022	2.51	1.14-5.51	0.022
	2024	1.96	0.93-4.10	0.076
Interaction: degree x survey year	Postgraduate x 2020	REF		
	Postgraduate x 2022	0.16	0.06-0.47	0.001
	Postgraduate x 2024	0.27	0.10-0.69	0.006

AOR, adjusted odds ratio; CI, confidence of interval; REF, reference group

Supplementary Table S7. Sensitivity analysis of factors associated with food insecurity among university students

		Multivariable[a]		
Variables	Category	AOR	95% CI	p-value
Student status	Domestic	REF		
	International	2.34	1.64-3.33	<0.001
	Other	1.56	0.51-4.71	0.434
Age group	<21 years	REF		
	21-25 years	1.38	0.95-2.01	0.093
	26-30 years	2.15	1.26-3.64	0.005
	>30 years	2.08	1.19-3.64	0.011
Gender	Female	REF		
	Male	1.53	1.12-2.09	0.007
Degree	Undergraduate	REF		
	Postgraduate	0.52	0.34-0.79	0.002
Survey year	2020	REF		
	2022	1.14	0.65-2.00	0.642
	2024	1.17	0.71-1.92	0.537
Employment status	Full time	REF		
	Part time	1.17	0.70-1.96	0.555
	Casual	0.97	0.58-1.60	0.892
	Unemployed	1.06	0.63-1.79	0.822
SEIFA IRSD	5 (least disadvantages)	REF		
	4	1.04	0.57-1.89	0.896
	3	1.13	0.69-1.87	0.620
	2	1.34	0.54-3.28	0.528
	1 (most disadvantages)	1.02	0.63-1.79	0.950
Household size	Alone	REF		
	2~3	0.70	0.42-1.18	0.187
	4~5	0.51	0.30-0.86	0.011
	6 or more	0.85	0.47-1.56	0.608

Sensitivity analysis used the single item measure in the Australian National Health Survey (McKechnie et al., 2018), which identified food insecurity when respondents affirm the statement. AOR, adjusted odds ratio; CI, confidence of interval; REF, reference group; SEIFA, socio-economic indexes for areas. SEIFA based on index of relative socio-economic disadvantage (IRSD): Q1 the most disadvantages and Q5 the least disadvantages.

[a] Adjusted for sociodemographic variables: student status, gender, age groups, education degrees, survey year, employment status, SEIFA, household size
